# Clusters of activated microglia in normal-appearing white matter show signs of innate immune activation

**DOI:** 10.1186/1742-2094-9-156

**Published:** 2012-07-02

**Authors:** Jack van Horssen, Shailender Singh, Susanne van der Pol, Markus Kipp, Jamie L Lim, Laura Peferoen, Wouter Gerritsen, Evert-Jan Kooi, Maarten E Witte, Jeroen JG Geurts, Helga E de Vries, Regina Peferoen-Baert, Peter J van den Elsen, Paul van der Valk, Sandra Amor

**Affiliations:** 1 Department of Molecular Cell Biology and Immunology/Neuropathology, VU University Medical Center, Van der Boechorststraat 7, 1081, BT, Amsterdam, The Netherlands; 2 Department of Pathology, VU University Medical Center, Amsterdam, The Netherlands; 3 Department of Neuropathology, University Medical Center, Georg-August University Göttingen, Göttingen, Germany; 4 Institute of Neuroanatomy, Faculty of Medicine, RWTH Aachen University, Wendlingweg 2, D-52074, Aachen, Germany; 5 Department of Anatomy and Neurosciences, VU University Medical Center, Amsterdam, The Netherlands; 6 Department of Immunohematology and Blood Transfusion, Leiden University Medical Center, Leiden, The Netherlands; 7 Neuroscience and Trauma Centre, Barts and the London School of Medicine and Dentistry, Queen Mary University of London, London, United Kingdom

**Keywords:** Multiple sclerosis, Microglial cells, Preactive lesion, Immune activation, Lesion development

## Abstract

**Background:**

In brain tissues from multiple sclerosis (MS) patients, clusters of activated HLA-DR-expressing microglia, also referred to as preactive lesions, are located throughout the normal-appearing white matter. The aim of this study was to gain more insight into the frequency, distribution and cellular architecture of preactive lesions using a large cohort of well-characterized MS brain samples.

**Methods:**

Here, we document the frequency of preactive lesions and their association with distinct white matter lesions in a cohort of 21 MS patients. Immunohistochemistry was used to gain further insight into the cellular and molecular composition of preactive lesions.

**Results:**

Preactive lesions were observed in a majority of MS patients (67%) irrespective of disease duration, gender or subtype of disease. Microglial clusters were predominantly observed in the vicinity of active demyelinating lesions and are not associated with T cell infiltrates, axonal alterations, activated astrocytes or blood–brain barrier disruption. Microglia in preactive lesions consistently express interleukin-10 and TNF-α, but not interleukin-4, whereas matrix metalloproteases-2 and −9 are virtually absent in microglial nodules. Interestingly, key subunits of the free-radical-generating enzyme NADPH oxidase-2 were abundantly expressed in microglial clusters.

**Conclusions:**

The high frequency of preactive lesions suggests that it is unlikely that most of them will progress into full-blown demyelinating lesions. Preactive lesions are not associated with blood–brain barrier disruption, suggesting that an intrinsic trigger of innate immune activation, rather than extrinsic factors crossing a damaged blood–brain barrier, induces the formation of clusters of activated microglia.

## Background

Multiple sclerosis (MS) is a chronic, progressive, inflammatory, demyelinating and neurodegenerative disease of the central nervous system (CNS). Examination of brain tissue of individuals with MS reveals focal lesions throughout the white matter and extensive demyelination in the cerebral cortex of chronic MS patients [1]. Common pathological features of MS white matter lesions include blood–brain barrier leakage, inflammatory infiltrates consisting of lymphocytes and macrophages, destruction of myelin sheaths and oligodendrocyte loss, axonal damage and loss, and glial scar formation [2-4]. In particular monocyte-derived macrophages and resident microglia contribute to MS lesion formation as they phagocytose myelin, leading to extensive myelin damage and oligodendrocyte dysfunction [5]. Importantly, during inflammation, activated microglia and infiltrated macrophages secrete various inflammatory mediators, including cytokines, chemokines, nitric oxide and reactive oxygen species (ROS), which all contribute to disease progression [5-7]. On the other hand it has been demonstrated that activated microglia and macrophages also exert beneficial effects by inducing remyelination, neuronal regeneration and repair [6,8,9]. Nonetheless, the initial trigger and molecular mechanisms underlying the pathogenesis of MS remain enigmatic.

Classification of MS lesions is usually based on standard histopathological staining for inflammatory cells and myelin proteins [10]. Active MS lesions are characterized by myelin loss and abundant phagocytic macrophages and microglia, which in some cases contain myelin degradation products. In time, these inflammatory lesions might transform into chronic active lesions with a hypocellular demyelinated gliotic center and a hypercellular rim containing myelin-laden macrophages and microglia. However, post-mortem material from patients with established MS mostly contain chronic inactive lesions, which are characterized by extensive astrogliosis in the absence of infiltrated immune cells and microglial activation. In some cases, remyelination is observed, indicating that intrinsic control of inflammation and repair does occur in the MS brain [11].

To date, the use of MRI-guided tissue sampling has significantly increased the yield of MS lesions in autopsy specimens. Particularly, lesions that are macroscopically invisible, including preactive lesions, are now commonly observed. In 2001, de Groot and colleagues described the occurrence of preactive lesions for the first time and speculated that this lesion type might represent one of the earliest stages of MS lesion development [12]. Preactive lesions are observed throughout the normal-appearing white matter and are characterized by clustering of activated microglia as demonstrated by enhanced expression of HLA-DR in the absence of overt demyelination. Many recent reports suggest that microglial activation represents an early stage of tissue damage, which precedes the formation of demyelinating lesions [2,13-16]. Although most histopathological studies have focused on active and chronic inactive lesions, remarkably little is known about the occurrence and cellular composition of preactive lesions [15]. The aim of this study was to gain more insight in the frequency, distribution and cellular architecture of preactive lesions using a large cohort of well-characterized MS brain samples.

## Methods

### Autopsy material

Brain tissue samples were obtained from the Netherlands Brain Bank (coordinator Dr. Huitinga, Amsterdam, The Netherlands). The Netherlands Brain Bank received permission to perform autopsies for the use of tissue and for access to medical records for research purposes from the Ethical Committee of the VU University Medical Center, Amsterdam, The Netherlands. All patients and controls, or their next of kin, had given informed consent for autopsy and use of brain tissue for research purposes. Relevant clinical information was retrieved from the medical records and is summarized in Table 1. A total of 213 paraffin-embedded tissue blocks from 21 MS patients (female-to-male ratio: 7:14; average age 59 years; average post-mortem delay 8 h) and 6 white matter blocks from 6 donors (female-to-male ratio: 4/2; average age 71; average post-mortem delay 7 h) without neurological disease were selected. Normal-appearing white matter (NAWM) and white matter lesion samples were selected on the basis of post-mortem MRI.

**Table 1:** Summary of patient details and lesion types

**MS cases**	**Age (years)**	**Type of MS**	**Gender**	**PM delay (h)**	**Disease duration (years)**	**No. tissue blocks**	**No. lesions**	**Preactive lesions**	**Active lesions**	**Chronic active lesions**	**Chronic inactive lesions**
1	63	PP	M	7	24	7	3	1	1	1	2
2	56	SP	M	8	21	7	5	2	0	2	0
3	69	ND	F	8	54	6	3	1	0	1	2
4	70	PP	F	7	40	8	3	2	0	1	2
5	78	SP	F	11	30	8	8	0	0	0	0
6	76	PP	F	10	19	7	5	0	0	0	2
7	47	ND	F	4	16	7	3	1	0	3	0
8	41	PP	M	7	14	9	1	8	2	5	0
9	66	ND	F	6	22	12	5	5	11	0	1
10	49	SP	M	8	25	17	5	1	0	11	0
11	55	PP	M	6	32	9	3	0	0	2	4
12	66	PP	M	8	26	14	1	5	0	13	0
13	57	SP	M	8	25	8	2	3	1	1	2
14	61	SP	M	9	41	14	7	2	0	5	1
15	45	SP	M	8	10	6	3	0	0	1	2
16	50	ND	M	10	16	10	4	0	0	1	5
17	44	PP	M	12	13	10	4	2	3	3	2
18	75	PP	M	8	39	8	7	0	0	1	1
19	68	PR	F	11	37	12	10	2	0	1	1
20	44	SP	M	10	22	17	3	9	0	10	0
21	51	PR	M	11	>10	17	5	0	6	4	1
						**213**	**90**	**44**	**24**	**66**	**28**
Controls											
1	57		M	6							
2	84		F	5							
3	72		M	10							
4	77		F	8							
5	72		F	5							
6	64		F	9							

PP, primary progressive; SP, secondary progressive; PR, progressive relapsing; ND, MS subtype not determined; F, female; M, male;

PM delay, post-mortem delay

### Immunohistochemistry

Formalin-fixed, paraffin-embedded tissue was sectioned at 5 μm and stained for proteolipid protein (PLP), HLA-DR (clone LN3) and CD68 as described previously [17]. Sections were deparaffinized in xylene and rehydrated through graded alcohol into distilled water. Endogenous peroxidase activity was quenched by incubating the slides in 0.3% hydrogen peroxide in methanol. For HLA-DR detection, slides were rinsed with distilled water and transferred to citric acid. Heat-induced antigen retrieval was performed using microwave irradiation for 5 min on the high setting and for 10 min on the medium setting. Slides were then cooled to room temperature and rinsed in phosphate-buffered saline (PBS). Serial sections were incubated overnight with anti-HLA-DR, anti-CD68 or PLP. Then, slides were incubated with EnVision Kit horseradish peroxidase-labeled anti-mouse/rabbit (DAKO) for 30 min at room temperature and finally diaminobenzidine tetrachloride. Between incubation steps, sections were thoroughly washed with PBS. After a short rinse in tap water, sections were incubated with hematoxylin for 1 min and extensively washed with tap water for 10 min. Finally, sections were dehydrated with ethanol followed by xylol and mounted with Entellan (Merck, Darmstadt, Germany). All antibodies were diluted in PBS containing 0.1% bovine serum albumin (Boehringer-Mannheim), which also served as a negative control. For the detection of NADPH oxidase-2 subunits gp91phox, p22phox and p47phox, we used cryosections and use the same Envision protocol as described above.

### Double immunohistochemistry

For double staining of amyloid precursor protein (APP) or CD3 with HLA-DR, sections were first stained for HLA-DR and labeled with DAB. Subsequently, APP and CD3 were detected with the alkaline phosphatase method. For double immunostaining for HLA-DR and FITC-labeled fibrinogen, claudin-5, glial fibrillary acidic protein (GFAP), neurofilament (NF), matrix metalloproteinase-2 (MMP2) and −9 (MMP9), tumor necrosis factor-α (TNF-α), interleukin-4 (IL4) and −10 (IL10), we used cryosections of snap-frozen brain tissue blocks (*n* = 10) from five MS patients (2 blocks per patient) with several preactive lesions (cases 8, 9, 12, 13 and 20). Cryosections (5 μm) were air-dried and fixed in acetone for 10 min and preincubated for 30 min with 20% goat serum, and subsequently incubated overnight at 4 °C with specific primary antibodies. Next, sections were incubated with either Alexa-546 or −594-labeled secondary antibodies (1:400; Molecular Probes) for 1 h at room temperature. Between the incubation steps, sections were thoroughly washed with PBS. After washing, slides were covered with Vectashield (Vector Laboratories) supplemented with 0.4% DAPI to stain nuclei. Microscopic analysis was performed with a Leica DM 6000 (Leica Microsystems, Heidelberg, Germany). Additional information on the antibodies used in this study is summarized in Table 2.

**Table 2:** Primary antibodies

**Primary antibody**	**Dilution**	**Company**
Proteolipid protein (PLP, clone plpc1)	1:500	Serotec Ltd.
HLA-DR (clone LN3)	1:75	eBioscience
CD 68 (clone KP1)	1:100	DAKO
gp91phox	1:100	[18]
p22phox	1:100	Santa Cruz
p47phox	1:50	Abcam
Amyloid precursor protein (APP)	1:2000	Millipore
CD3	1:100	DAKO
Glucose transporter-1	1:100	Abcam
FITC-labeled fibrinogen	1:100	DAKO
Claudin-5	1:100	Invitrogen
Glial fibrillary acidic protein (GFAP)	1:20	Monosan
Neurofilament (NF)	1:100	Abcam
Matrix metalloproteinase-2 (MMP2)	1:100	Abcam
Matrix metalloproteinase-9 (MMP9)	1:500	Calbiochem
Tumor necrosis factor-α (TNF-α)	1:200	Acris
Interleukin-4 (IL4)	1:70	R&D Systems
Interleukin-10 (IL10)	1:100	R&D Systems

### Statistics

Numbers of blocks containing different lesions types were counted separately for all patients included in the study and expressed as percentage. Pearson correlation testing was performed for respective groups. The frequency of preactive lesions relative to other white matter lesions was compared using one-way ANOVA followed by Tukey’s multiple comparison test.

## Results

### Frequency and distribution of preactive lesions in MS brain tissue

**Figure 1:**
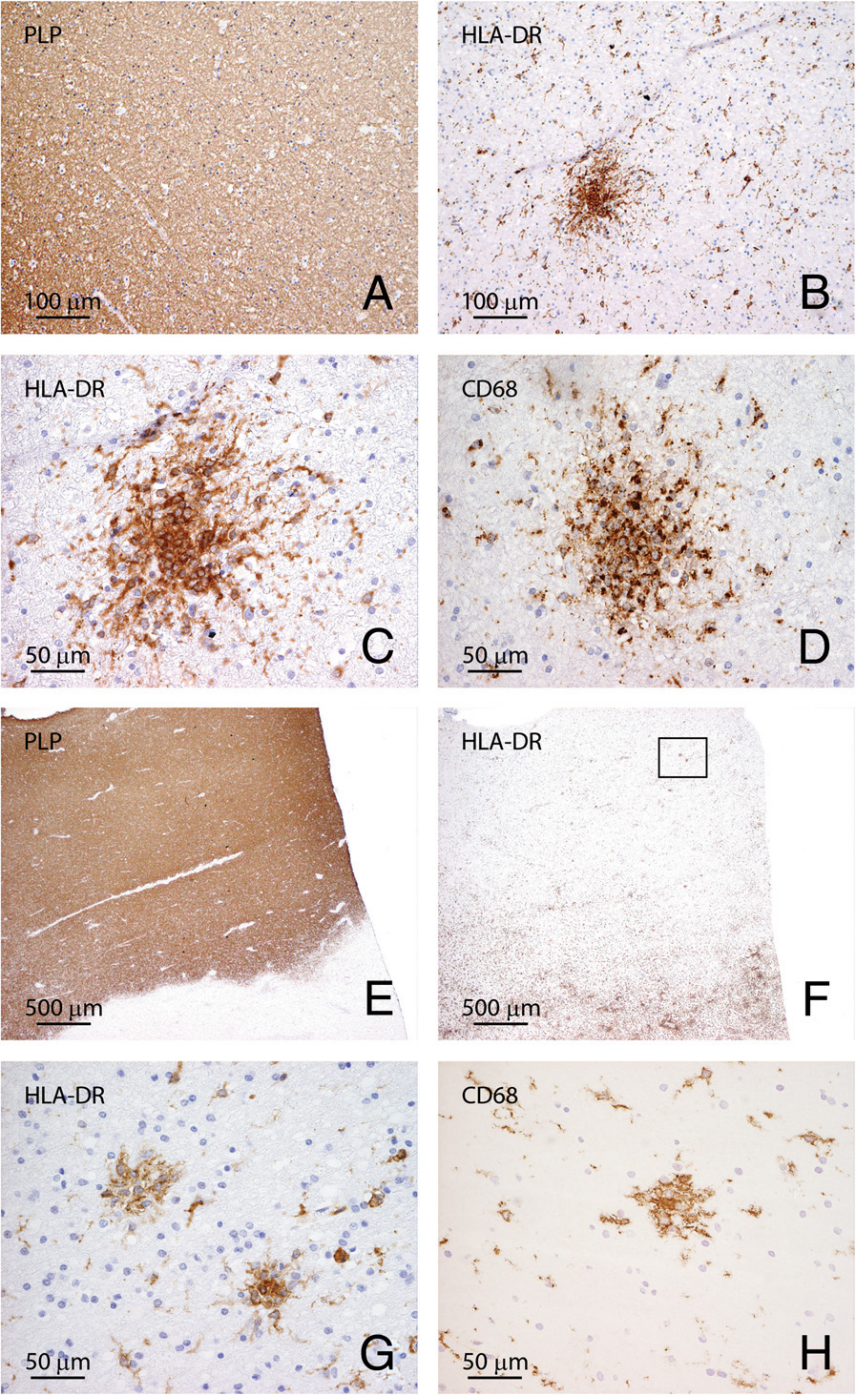
**Preactive lesions are composed of clusters of HLA-DR-positive microglia.** In NAWM, i.e., in the absence of apparent myelin loss (**A**, **E**: proteolipid protein) preactive lesions are defined as circumscribed nodules of activated microglia expressing HLA-DR (**B**, **C**) and CD68 (**D**). Preactive lesions are predominantly observed in blocks containing active lesions (**E**: proteolipid protein; **F**, **G**: HLA-DR). Figure 1G represents a magnification of the outlined square in (F). In some cases microglial nodules are surrounded by a halo devoid of microglia (H: HLA-DR). Original magnifications: A, B: 20×; C, D: 40×; E, F: 4×; G, H: 40×.

To gain insight into the frequency of preactive lesions, we randomly selected a cohort of 21 MS patients from our database between 2006 and 2010. Of these 21 MS patients, a total of 213 tissue blocks were collected. Identification of lesions was based on immunohistochemical staining for microglia/macrophages (anti-HLA-DR) and proteolipid protein (PLP). From the total number of tissue blocks, we observed 24 blocks with active lesions with severe myelin loss and abundant phagocytic macrophages and microglia, 66 blocks with chronic active lesions with a demyelinated center and a border containing macrophages and microglia, and 28 blocks with chronic inactive lesions with few HLA-DR-positive inflammatory cells and extensive astrogliosis in 213 tissue samples derived from 21 MS cases (for patient details and lesion distribution, see Table 1). Preactive lesions, defined as circumscribed nodules of activated microglia (HLA-DR + and CD68+) in the absence of apparent myelin loss (Figure 1), were observed in 44 out of these 213 tissue blocks (21%) from 14 MS patients (67%) and absent in all white matter samples from non-neurological control patients. Occasionally, we observed preactive lesions that were surrounded by a halo devoid of HLA-DR-positive microglia, suggesting that microglia from this area had migrated towards the ‘center’ of the preactive lesion (Figure 1H). Preactive lesions were found in both male (9) and female (5) MS patients irrespective of MS subtype (Table 1). Notably, preactive lesions were observed in both patients with relatively short disease duration, e.g., cases 8 (14 years) and 17 (13 years), and patients with long disease duration, e.g., cases 3 (54 years) and 4 (40 years) (Table 1).

**Figure 2:**
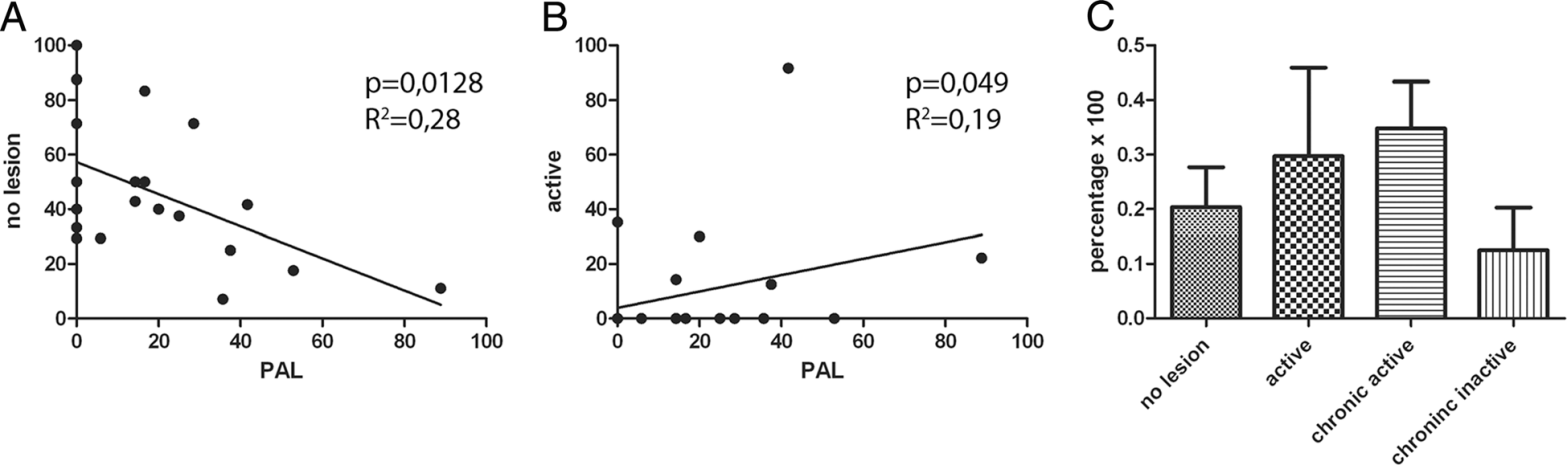
**Correlation of preactive lesions with the occurrence of white matter lesions.** Presence of preactive lesions (PAL) in tissue blocks in NAWM (**A**) or blocks containing active and chronic active lesions (**B**). The numbers of blocks of NAWM (**A**) or active lesions (**B**) were expressed as percentage of all blocks. Pearson correlation testing was performed for respective groups. The presence of preactive lesions negatively correlates with the number of blocks containing no lesions (*p* = 0.0128; R^2^ 0.28), whereas the presence of preactive lesions positively correlates with the number of active lesions (*p* = 0.049; R^2^ 0.19). Frequency of preactive lesions were determined in respective lesions and results compared using one-way ANOVA followed by Tukey’s multiple comparison test. A tendency exists that preactive lesions are more frequently observed in blocks containing active and chronic active lesions compared to blocks containing no lesion or chronic inactive lesions; however, this failed being statistically significant (**C**).

Next, we studied the association between preactive lesions and inflammatory white matter lesions. The occurrence of preactive lesions negatively correlates with the number of blocks containing no active lesions (Figure 2A; *p* = 0.0128), whereas the presence of preactive lesions weakly correlates with the number of blocks with active lesions (Figure 2B; *p* = 0.049). No significant correlation was found between the presence of preactive lesions and the number of chronic active lesion or chronic inactive lesion blocks (data not shown). Statistical analysis revealed that the occurrence of preactive lesions is not associated with the different types of white matter lesions within the same block, and preactive lesions were even detected in tissue sections of blocks lacking white matter lesions (Figure 2C). Taken together, our observations reveal that preactive lesions, characterized by clusters of activated microglia, can be observed in tissue blocks containing different stages of white matter lesions, but have a tendency to occur more frequently in the vicinity of active lesions. Moreover, preactive lesions are present in MS brain tissue samples regardless of gender, type of MS or disease duration.

### Cellular composition of preactive lesions

**Figure 3:**
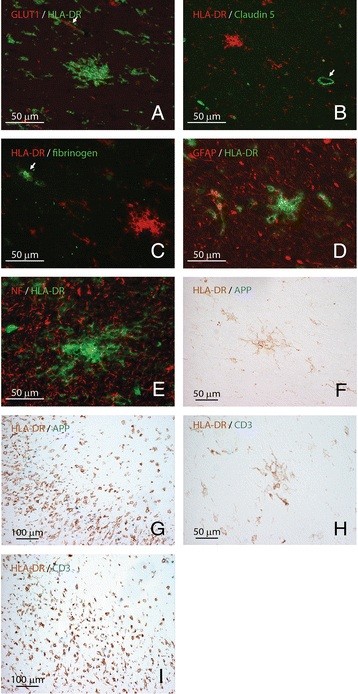
**Cellular organization of preactive lesions.** Preactive lesions were not associated with the cerebrovasculature (A: HLA-DR in green, glucose transporter protein-1 in red, arrows indicate vessels). No alterations were observed in the expression of the tight junction molecule claudin-5 (B: HLA-DR in red, claudin-5 in green, arrow indicates vessel), and fibrinogen immunoreactivity (C: HLA-DR in red, fibrinogen-FITC in green) was restricted to the lumen of the cerebrovasculature in areas with preactive lesions (C, arrow). Analysis on the expression of glial fibrillary acidic protein (D: HLA-DR in green, glial fibrillary protein in red) and neurofilament (E: HLA-DR in green, neurofilament in red), common markers for astrocytes and axons, respectively, demonstrated no signs of astrogliosis or axonal changes in or surrounding preactive lesions. Intra-axonal amyloid precursor protein accumulation, indicative of acute axonal injury, was absent in preactive lesions (F: APP in blue, HLA-DR in brown), but present in the rim of a chronic active MS lesions (G: APP in blue, HLA-DR in brown). T cell infiltrates were not associated with preactive lesions (H: CD3 in blue, HLA-DR in brown), but restricted to highly inflammatory areas (I: CD3 in blue, HLA-DR in brown). Original magnifications: A, C-F and H: 40×; B, G and I: 20×.

To examine the cellular composition of preactive lesions, we performed double immunohistochemistry on ten tissue blocks from five cases with abundant preactive lesions (cases 8, 9, 12, 13 and 20) using markers for different CNS cells and typical pathological features of MS pathology. Since MS lesions are generally concentrated around inflamed venules and veins, we first analyzed the association between preactive lesions and the cerebrovasculature, and showed that preactive lesions are not evidently associated with blood vessels (Figure 3A). Moreover, markers for blood–brain barrier disruption, including the key tight junction molecule claudin-5 (Figure 3B) and fibrinogen (Figure 3C), a serum component that is commonly used as a marker for leaky vessels, revealed no marked changes in the blood–brain barrier integrity surrounding microglial clusters. Analysis of the expression of glial fibrillary acidic protein (Figure 3D) and neurofilaments (Figure 3E), common markers for astrocytes and axons, respectively, demonstrated no signs of astrogliosis or axonal changes in or surrounding preactive lesions. Intra-axonal amyloid precursor protein accumulation, indicative of acute axonal injury, was absent from preactive lesions (Figure 3F), but present in the rim of a chronic active MS lesions (Figure 3G). T cell infiltrates were not associated with preactive lesions (Figure 3H), but restricted to highly inflammatory areas (Figure 3I).

### Preactive lesions express distinct inflammatory mediators

**Figure 4:**
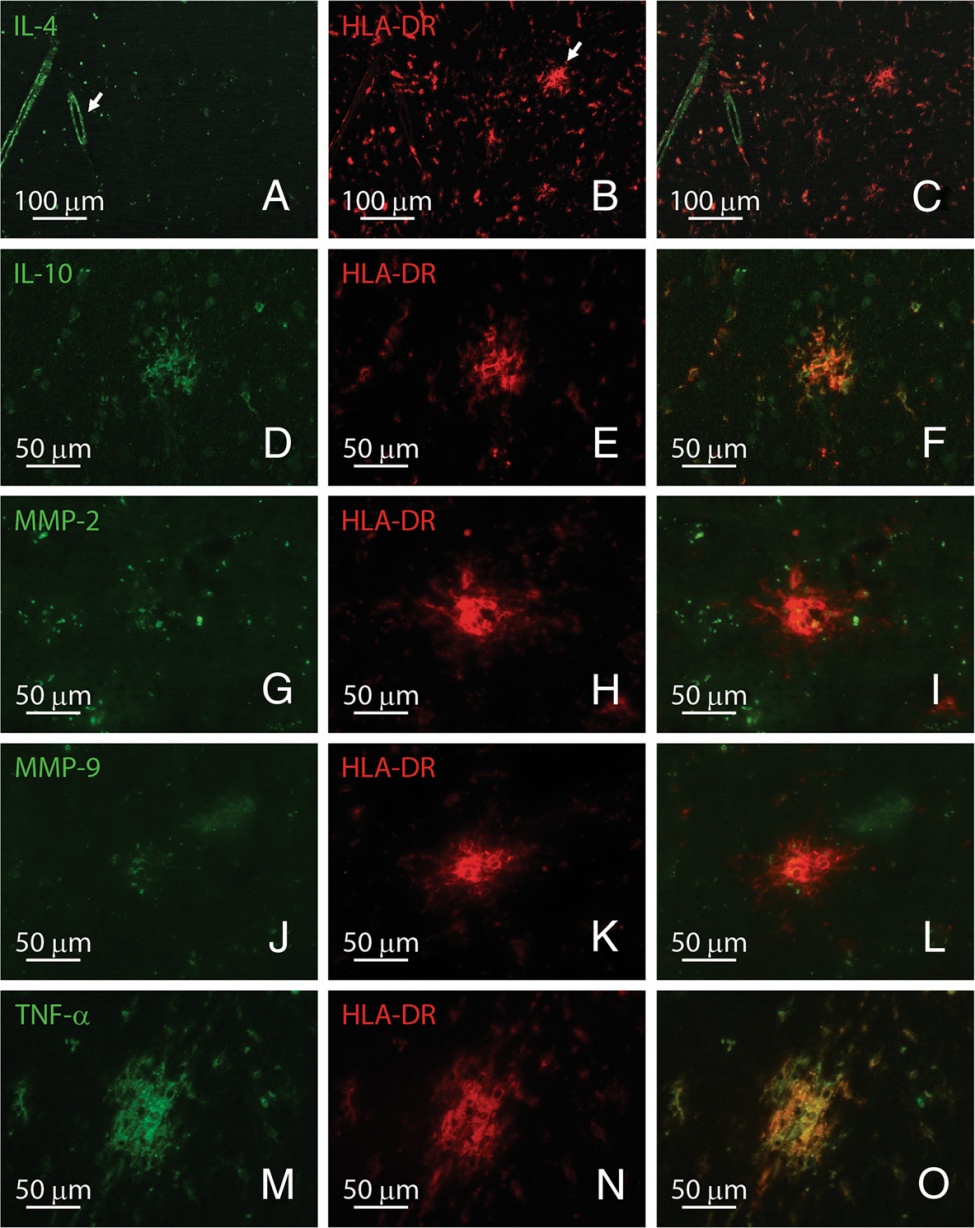
**Microglia associated with preactive lesions express distinct inflammatory mediators.** Interleukin-4 is present in the cerebrovasculature (A) and not in preactive lesions (B, arrow), (A-C; interleukin-4 in green, HLA-DR in red). The cytokine interleukin-10 was expressed by microglia in preactive lesions (d-F; interleukin-10 in green, HLA-DR in red). Both matrix metalloproteinase-2 (G-I; MMP-2 in green, HLA-DR in red) and −9 (J-L; MMP-9 in green, HLA-DR in red) were virtually absent in preactive lesions. TNF-α was strongly expressed by microglia in preactive lesions (M-O; TNF-α in green, HLA-DR in red). Original magnifications A-C: 10×; d-O: 40×.

To examine the phenotype of microglia in preactive lesions, we determined the expression of several key molecules known to be involved in MS pathology in the same tissue blocks used to study the cellular composition of preactive lesions. Interleukin-4 immunostaining was consistently observed in the cerebrovasculature and not associated with microglia (Figure 4A-C), whereas clusters of activated microglia markedly expressed interleukin-10 (Figure 4D-F). Matrix metalloproteinase-2 (Figure 4G-I) and −9 (Figure 4J-L) immunostaining, both involved in the breakdown of extracellular matrix, was virtually lacking in nodules of activated microglia, whereas TNF-α was clearly localized to microglia in preactive lesions (Figure 4M-O).

### Increased microglial NADPH oxidase-2 immunostaining in preactive lesions

**Figure 5:**
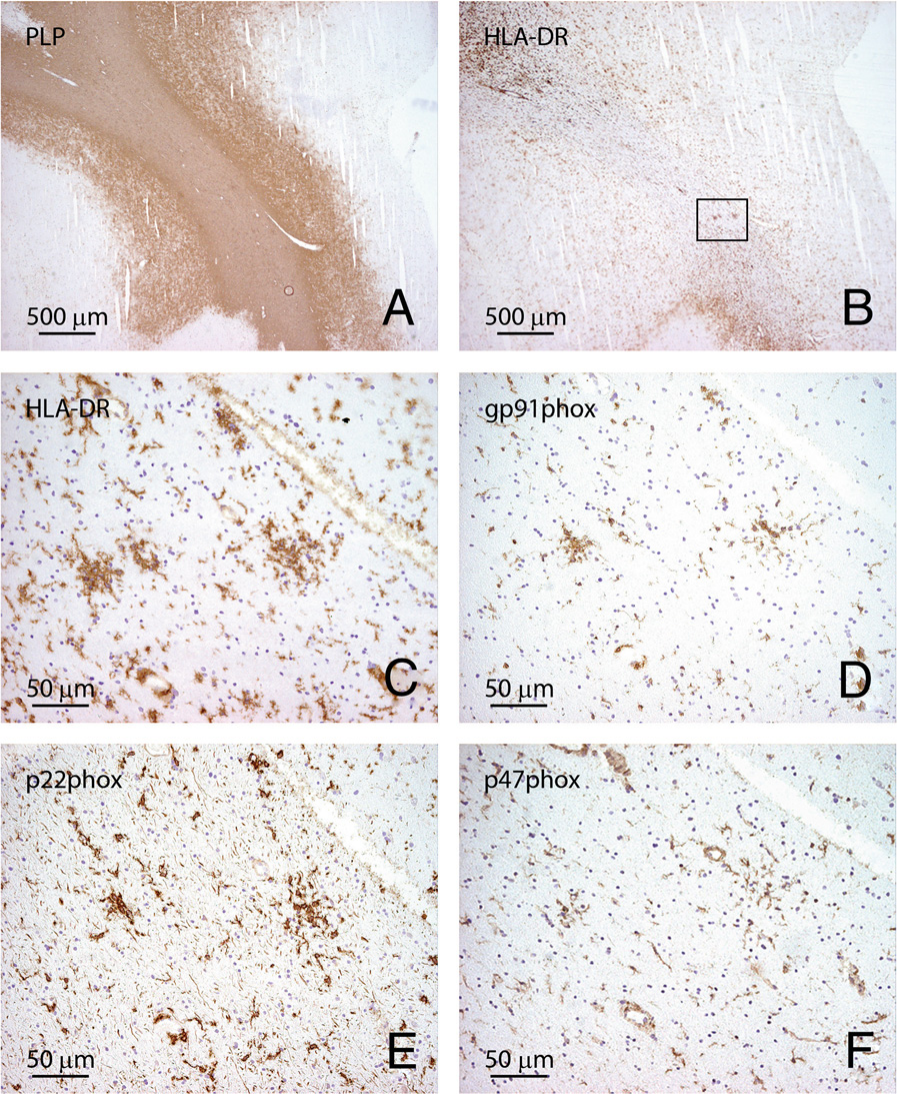
**Microglia associated with preactive lesions express NADPH oxidase-2 subunits.** Clusters of HLA-DR immunopositive microglia (**B** and **C**, HLA-DR) in NAWM (PLP, **A**) express various NADPH oxidase-2 subunits, including gp91phox (**D**), p22phox (**E**) and p47phox (**F**) in consecutive sections. Figure 1C-F represents a magnification of the outlined square in Figure 1B. Original magnifications A, B: 4×, C-F: 40×.

Recently, we demonstrated that various subunits of the ROS-generating enzyme NADPH oxidase-2 are clearly upregulated in activated microglia in active demyelinating and slowly expanding chronic MS lesions [19]. Here, we analyzed the expression of three key NADPH oxidase-2 subunits, namely gp91phox, p22phox and p47phox, in the same tissue blocks as described above. Preactive lesions, defined as microglial clusters in NAWM [Figure 5A (PLP), B,C (HL-DRA)], were consistently labeled with antibodies directed against the NADPH oxidase-2 subunits, gp91phox (Figure 5D), p22phox (Figure 5E) and p47phox (Figure 5F) in consecutive sections.

## Discussion

Despite major advances in the development of novel MS therapeutics that significantly reduce the number of exacerbations, the etiology of this chronic and devastating disease still remains unclear. Most therapeutic approaches are effective in the initial inflammatory phase of the disease, but fail to limit the ongoing neurodegeneration. Consequently, the concept that MS is a purely immune-mediated, or indeed autoimmune-mediated disease, has been challenged [20]. Despite these ideas and many comprehensive neuropathological studies, little is known about the initial trigger of focal MS lesions.

The NAWM represents an interesting region to search for clues that might trigger the formation of a demyelinating lesion [13]. Several neuropathological, biochemical and imaging studies reveal alterations in the NAWM, including signs of oligodendrocyte stress, subtle myelin changes and axonal alterations [16,21-25]. Perhaps the most prominent pathological feature in NAWM is the occurrence of widespread microglial activation and clustering [26]. The detection of nodules of strongly positive HLA-DR-positive activated microglia in the absence of overt demyelination in NAWM of MS patients has led to the classification of so-called preactive lesions [12]. Here, we examined the cellular composition and immune markers expressed in preactive MS lesions, and their relationship with focal demyelinated white matter lesions. Preactive lesions were frequently observed in 14 out of 21 MS patients (67%), which is in line with previous observations by De Groot and colleagues [12]. The frequency of preactive lesions was higher, albeit weakly significantly, in regions in the vicinity of active lesions. Preactive lesions were observed in the majority of patients with established MS, suggesting that their occurrence is not restricted to the early phase of the disease, but that focal clusters of activated microglia continuously emerge regardless of disease duration. Since preactive lesions were frequently detected even in patients with long-standing disease, it is unlikely that all preactive lesions eventually progress into demyelinating lesions. Instead, these observations suggest that most of them are likely to spontaneously resolve over time without progressing into an active demyelinating stage.

Microglia are innate immune cells of myeloid origin residing in the CNS and continuously screen their surroundings. They react rapidly to changes in their microenvironment, and can become morphologically and functionally activated by a variety of triggers. Activated microglia in MS brains can be detrimental by amplifying an inflammatory response by the production of cytokines, chemokines and free radicals, which might contribute to oligodendrocyte cell death, axonal injury and demyelination. Alternatively, activated microglia produce factors that are known to promote remyelination and stimulate neuroprotection. This study reveals that microglia in preactive lesions express TNF-α and IL-10, whereas preactive lesions were virtually devoid of IL-4, and MMP-2 and −9. TNF-α is a cytokine that exerts pleiotropic functions, and although it is usually considered as a proinflammatory mediator, it also activates cell survival pathways (reviewed in [27]). Thus, the presence of TNF-α, as well as IL-10, suggests attempts to regulate inflammation. We recently described enhanced NADP oxidase expression in activated microglia in active and chronic active MS lesions, indicating the important role of free radicals in demyelination and associated tissue injury [19]. In the current study, we demonstrate that microglia in preactive lesions also show pronounced expression of key subunits of NADPH oxidase-2, indicating the assembly of a functional NADPH oxidase-2 complex. This strongly suggests that activated microglia in preactive lesions might be involved in the production of reactive oxygen species.

Although the white matter is highly vascularized, preactive lesions did not clearly localize to blood vessels and microvessels in close vicinity to preactive lesions showed no clear signs of tight junction alterations. In addition, we observed no extravascular deposition of the serum protein fibrinogen or infiltrated T cells, indicating the lack of blood–brain barrier disruption. The apparently intact blood–brain barrier in preactive lesions suggests that a yet-unknown intrinsic CNS trigger induces the microglial activation and clustering rather than a response to T cells or serum components that enter via a compromised blood–brain barrier. Potential pathogenic triggers of microglial activation and clustering in the NAWM might be axonal injury or oligodendrocyte stress. Microarray analysis has revealed that both anti-inflammatory as well as pro-inflammatory genes are consistently upregulated in NAWM, particularly in oligodendrocytes [24]. In search for early molecular alterations in NAWM, Zeis and colleagues identified oxidative damage to oligodendrocytes, which was paralleled by an increase in the expression of genes known to be involved in antioxidant defense [23,28]. In addition, we previously reported the presence of αB-crystallin-immunopositive oligodendrocytes in preactive lesions, indicative of oligodendrocyte stress [29]. Importantly, neurofilament staining showed a normal expression pattern, and APP-positive axonal bulbs were not associated with preactive lesions, but restricted to areas with abundant inflammatory cells. Nonetheless, we cannot exclude the possibility that more subtle axonal alterations, such as changes in axonal ion transporters or mitochondrial dysfunction [30-33], lead to microglial activation and clustering.

## Conclusion

Taken together, we showed that preactive lesions are detected in the majority of MS samples irrespective of gender, disease duration or subtype of disease. Given the high frequency of preactive lesions in our set of MS brain samples, it appears unlikely that most of them will progress into full-blown demyelinating lesions. The balance between proinflammatory signals, such as reactive oxygen species, and anti-apoptotic signals or anti-inflammatory signals, e.g., IL-10, may be the determining factor in lesion development. Although the number of preactive lesions was higher in areas surrounding inflammatory lesions, microglial clusters were not associated with T cell influx, axonal changes or activated astrocytes. Importantly, vessels in close proximity to preactive lesions showed no signs of blood–brain barrier disruption, suggesting that an intrinsic trigger of innate immune activation rather than extrinsic factors crossing a damaged blood–brain barrier induce the formation of clusters of activated microglia.

## Competing interest

The author(s) declare that they have no competing interests.

## Authors’ contributions

JvH and SS analyzed the data and wrote the manuscript. SvdP, JL, LP, WG and RPB carried out the immunohistochemical analyses. MK and MW participated in the design, analyzed the data and prepared the figures. E-JK, JG, HdV, PvdE and PvdV reviewed the manuscript and SA supervised the study. All authors read and approved the final manuscript.
